# Relevance of circulating nucleosomes and oncological biomarkers for predicting response to transarterial chemoembolization therapy in liver cancer patients

**DOI:** 10.1186/1471-2407-11-202

**Published:** 2011-05-26

**Authors:** Nikolaus Kohles, Dorothea Nagel, Dietrich Jüngst, Jürgen Durner, Petra Stieber, Stefan Holdenrieder

**Affiliations:** 1 Institute of Clinical Chemistry, University-Hospital Munich-Grosshadern, Germany; 2 Medical Clinic II, University-Hospital Munich-Grosshadern, Germany; 3 Institute of Clinical Chemistry and Clinical Pharmacology, University Hospital Bonn, Germany

## Abstract

**Background:**

Transarterial chemoembolization (TACE) therapy is an effective locoregional treatment in hepatocellular cancer (HCC) patients. For early modification of therapy, markers predicting therapy response are urgently required.

**Methods:**

Here, sera of 50 prospectively and consecutively included HCC patients undergoing 71 TACE therapies were taken before and 3 h, 6 h and 24 h after TACE application to analyze concentrations of circulating nucleosomes, cytokeratin-19 fragments (CYFRA 21-1), alpha fetoprotein (AFP), C-reactive protein (CRP) and several liver biomarkers, and to compare these with radiological response to therapy.

**Results:**

While nucleosomes, CYFRA 21-1, CRP and some liver biomarkers increased already 24 h after TACE, percental changes of nucleosome concentrations before and 24 h after TACE and pre- and posttherapeutic values of AFP, gamma-glutamyl-transferase (GGT) and alkaline phosphatase (AP) significantly indicated the later therapy response (39 progression versus 32 no progression). In multivariate analysis, nucleosomes (24 h), AP (24 h) and TACE number were independent predictive markers. The risk score of this combination model achieved an AUC of 81.8% in receiver operating characteristic (ROC) curves and a sensitivity for prediction of non-response to therapy of 41% at 97% specificity, and of 72% at 78% specificity.

**Conclusion:**

Circulating nucleosomes and liver markers are valuable tools for early estimation of the efficacy of TACE therapy in HCC patients.

## Background

Hepatocellular carcinoma (HCC) is the fifth most common cancer worldwide and the third most common cause of cancer-related death with approximately 500,000 deaths each year [1]. In recent years, incidence of HCC has been increasing in western countries [2]. The major risk factors of HCC are liver cirrhosis, alcohol abuse, and hepatitis B and C virus infections [3-5]. Most patients have at least two concomitant diseases, such as chronic liver disease and HCC, while complex interactions between these have major implications for diagnosis, prognosis and management of HCC. The clinical course of patients is determined by both liver function and the extent of HCC. Curative therapy options include resection, liver transplantation and radiofrequency ablation (RFA). However, these are often not applicable due to advanced tumor stage [6,7].

The best therapy option available for HCC patients who cannot be treated curably is transarterial chemoembolization (TACE), where iodized oil mixed with anticancer agents and an embolic material is administered through the hepatic artery. Iodized oil is used as a vehicle to transport the anticancer drug, while the embolic material enhances the antitumor effect of the anticancer drug. Normal liver tissue is spared as blood supply is mainly delivered via the portal vein, whereas the tumor cells are mainly nourished by the hepatic artery. Therefore, TACE is a minimally invasive procedure with a demonstrated ability to reduce systemic toxicity, increase local effects and improve overall therapeutic results, particularly in the treatment of unresectable hepatocellular carcinoma. Thus, the approach of TACE is a palliative treatment to control tumor growth [8-13].

In recent studies, some parameters, such as tumor size, portal invasion, alpha fetoprotein (AFP), Child-Pugh score, bilirubin, ascites, and performance status were found to have prognostic and predictive impact if determined prior to TACE [14]. Yet, there are still no accepted parameters to evaluate the early therapy response after TACE. However, these predictive and prognostic markers are important as the early modification of the treatment strategy could potentially save time as well as costs. Recently, various techniques, such as computed tomography (CT) or magnetic resonance imaging (MRI), have demonstrated to be able to indicate insufficient response to therapy within one or two month [15-17]. In addition, the course of the apparent diffusion coefficient (ADC) in MRI before and after TACE is supposed to monitor therapy response within several days after treatment [18,19]. However, further easily obtainable sensitive and robust markers are required to efficiently monitor TACE treatment. Most promising candidates are biochemical markers circulating in the blood that are related to the disease and therapy as they can be measured non-invasively and cost-effectively, also in serial determinations.

In lung cancer, cell death markers, such as nucleosomes and cytokeratin-19 fragments (CYFRA 21-1), have been identified as relevant biochemical markers enabling early estimation of systemic chemotherapy efficacy [20,21]. In the present study, we investigated these markers, the HCC-associated marker AFP and other liver enzymes to identify pre- or post-treatment variables that may anticipate therapy response already in the early treatment phase.

## Methods

### Patients

In the present study cohort, 50 patients suffering from hepatocellular cancer (42 males and eight females, mean age 66.7 years) were prospectively and consecutively included. All patients were diagnosed and staged at the University Hospital Munich-Grosshadern between 2006 and 2008. Patient characteristics are summarized in Table 1 (Table 1). Diagnosis of HCC was confirmed by fine needle biopsy or radiological criteria (two coincident imaging techniques) or combined criteria (one imaging technique associated with elevated AFP levels according to the Barcelona EASL Conference 2000 criteria) [22]. The study was approved by the local ethics committee and written informed consent was obtained from each patient before first blood sampling.

**Table 1:** Characteristics of patients with hepatocellular cancer

	Median	Range
**Age**	66.7	45.1 - 83.7
**Survival**	14.5	1.1 - 25.1

	**Number**	**Percentage (%)**

***Total***	**50**	100
**Gender**		
Female	8	16.0
Male	42	84.0
**Cirrhosis**	37	74.0
**Child-Pugh stage**		
A	20	40.0
B	14	28.0
C	3	6.0
**Risk factors**		
Hepatitis B positive	2	4.0
Hepatitis C positive	8	16.0
HBV + HCV	3	6.0
Alcohol-induced cirrhosis	21	42.0
Alcohol + HCV	1	2.0
**Number of tumors**		
single lesion	13	26.0
double lesions	8	16.0
multiple lesions	29	58.0
**Tumor size**		
≤ 1 cm	2	4.0
≤ 3 cm	14	28.0
≤ 5 cm	17	34.0
≤ 10 cm	13	26.0
> 10 cm	4	8.0
**T-stage**		
T1	10	20.0
T2	4	8.0
T3	36	72.0
**Treatments before TACE**		
Resection	5	10.0
PEI	2	4.0
**Indication of TACE**		
Palliative	33	66.0
Bridging	5	10.0
bridging (plus RFA)	1	2.0
palliative (plus RFA)	5	10.0
palliative after prior resection	5	10.0
**Treatments after TACE**		
TACE	21	42.0
Sorafenib	7	14.0
TACE and Sorafenib	4	8.0
LTX	6	12.0
**Time of study entry**		
1st application	21	42.0
2^nd^-further application	29	58.0

**Total therapies**	**77**	100

**Therapy**		
TACE	71	92.2
TACE + RFA	6	7.8
**Number of TACE cycle**		
1	21	29.6
2	20	28.2
3	16	22.5
4	7	9.9
≥5	7	9.9
**Time of CT- and MRI-Scan after TACE**		
< 60 days	20	28.2
60 - 90 days	36	50.7
> 90 days	15	21.1
**Therapy response (TACE alone)**		
REM	2	2.8
SD	30	42.3
PD	39	54.9

### Treatment procedures

All TACE procedures were performed under angiographic control (Multistar TOP and Axiom Artis dTA, Siemens, Munich, Germany) and local anesthesia. After inserting a 4-French (Fr) pigtail catheter into the femoral artery via micro incision into the groin, panviscerography was performed to detect potentially aberrant or additional hepatic and possibly tumor-feeding arteries. After identifying the tumor-feeding arteries, a 4-Fr catheter (e.g. cobra configuration) for selective use or a superselective microcatheter, which could be placed through the primary 4-Fr catheter, were directed as close as possible to the tumor-feeding vessels. The embolizing moiety was prepared by extensive mixing between two syringes, typically consisting of 3-5 ml lipiodol, microparticles of 150-500 μm (e.g. Contour SE^®^, Boston Scientific, Ratingen, Germany) and farmorubicin (1 mg/kg b.w.). Subsequently, the embolizing agent was slowly injected under fluoroscopic control to avoid retrograde embolization of non-target areas due to back spill. The injection was stopped as soon as stasis within the tumor vessels occurred. Treatment was terminated if a flow within the tumor vessels was no longer detectable after 5-10 min. Otherwise, another injection was performed. In case of several main feeders, vessels were treated subsequently.

### Classification of response to therapy

In all patients, staging investigations, consisting of abdominal CT or MRI and laboratory examinations, were performed prior to the next TACE therapy. The median evaluation time of therapy response was 70 days (range: 22 - 216 days) after TACE. The response to therapy was classified according to RECIST criteria for solid tumors defining 'complete remission' as complete disappearance of all such manifestations of disease, 'partial remission' as reduction of tumor diameter ≥ 30%, 'progression' as tumor increase ≥ 20% or appearance of new tumor manifestations, and 'stable disease' as tumor reduction < 30% or increase < 20% in medical imaging [23].

### Sample collection and assays

Blood samples were collected prospectively prior to TACE, and three, six and 24 hours (h) after TACE. We performed the preanalytical handling of the samples as described in Holdenrieder et al. [24]: Blood samples were centrifuged at 3,000 × G for 15 min within one to two hours after venipuncture. Subsequently, serum samples were stabilized by adding 10 m*M *EDTA and stored at -80°C. Prior to measurement of nucleosomes, samples were thawed, homogenized and diluted 1:4 with an incubation buffer. The courses of nucleosomes of single patients were determined within one run of the enzyme immunoassay to minimize methodical variance.

Quantification of nucleosome concentrations in serum was done by use of Cell Death Detection Elisa^plus ^of Roche Diagnostics (Mannheim, Germany). Two monoclonal mouse antibodies, directed against histones and DNA, respectively, specifically catch the nucleosomes. While the anti-histone antibodies fix the complexes to the microtiter plate, the anti-DNA antibodies, which are labeled with peroxidase, react with the added 2,2'-azino-di-(3-ethylbenzthiazolin-sulfonate) substrate. The resulting color development is proportional to the amount of nucleosomes captured in the antibody sandwich. This enables photometric quantification of nucleosomes in ng/mL according to an established standard curve.

In addition, we determined CYFRA 21-1 and AFP by Elecsys 2010 (Roche Diagnostics, Mannheim, Germany) and lactate dehydrogenase (LDH), C-reactive protein (CRP), aspartate-aminotransferase (AST), alanine-aminotransferase (ALT), glutamate dehydrogenase (GLDH), bilirubin, gamma-glutamyl-transferase (GGT), alkaline phosphatase (AP), amylase, lipase and choline esterase (CHE) by high-end analyzer AU 2700 (Olympus Diagnostics, Hamburg, Germany) in all serum samples.

### Statistics

Concentrations of all measured markers prior to and three, six and 24 hours after TACE as well as their percental changes were considered for statistical evaluation.

Concerning their response to therapy, partial remission and stable disease were assigned to the 'no progression' group. They were compared with patients who suffered from progressive disease. As six TACE treatments were accompanied by RFA treatment the following day these were excluded from the evaluation of therapy response due to confounding effects.

Wilcoxon test was applied for comparison of marker concentrations between the two groups in relation to therapy response. The influence of TACE cycle number on marker values (cycle 1 vs cycles ≥2; and cycles 1+2 vs cycles > 3) was also calculated by Wilcoxon test; influence of TACE cycle number on therapy response was tested by Mantel-Haenszel-Chi-Square test; association of T-stage (T1+2 vs T3) on therapy response was analyzed by Chi-Square test. Univariately relevant markers indicating therapy response (after logarithmizing to the basis 2) and clinical factors were included in a multivariate logistic regression analysis to identify the best prediction model.

A *p*-value of < 0.05 was considered statistically significant. All calculations were performed with SAS software (version 9.2, SAS Institute Inc., Cary, N.C., USA).

## Results

In the present study, 50 patients with a total of 77 TACE treatments were evaluated. After exclusion of six treatments due to subsequent RFA treatment, 71 treatments were evaluated for their response to TACE according to staging by imaging after approximately two months (median 70 days). In these imaging investigations, 32 TACE-treatments out of 71 showed stable disease (N = 30) or partial remission (N = 2) after TACE (no progression), while the remaining 39 treatments showed progressive disease. In 21 cases, first cycle of TACE was applied, while in 50 cases, the second or later TACE cycles were applied. Details of patient characteristics are listed in Table 1 (Table 1).

### Kinetics of the markers

**Figure 1:**
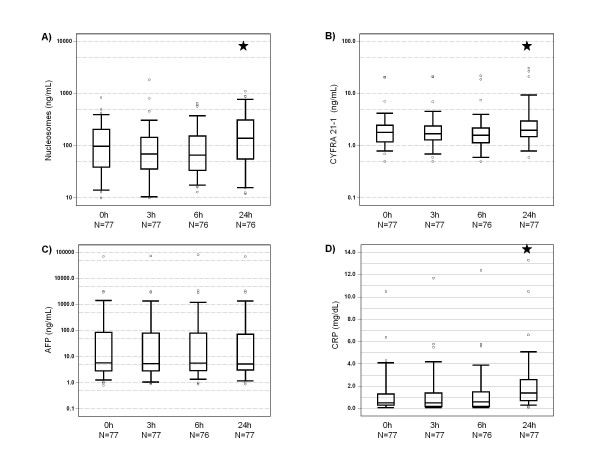
**Box-plots of serum levels of nucleosomes, cytokeratin-19 fragments (CYFRA 21-1), alpha fetoprotein (AFP), and C-reactive protein (CRP) showing medians, interquartile ranges, minimum and maximum in all patients during the first day after TACE (★ indicate significant changes from 0 h values)**.

As early as 24 h after application of TACE, concentrations of circulating nucleosomes, CYFRA 21-1, LDH, CRP, bilirubin, and activity of liver enzymes were increased in most patients. In contrast, median activities of pancreas enzymes lipase, amylase and alkaline phosphatase (AP), and choline esterase, as well as concentration of AFP were decreased or stable when compared to pretherapeutic values (Table 2 and Figure 1).

**Table 2:** Levels of biomarkers before, 3 h, 6 h, and 24 h after application of TACE (medians; ranges; p-values for changes from 0 h)

	0 h	3 h	6 h	24 h
**Nucleosomes (ng/mL)**	97.8	69.8	66.4	139.3
	(10.0 - 846.0)	(10.0 - 1861.3)	(13.0 - 660.1)	(11.9 - 1116.5)
		0.067	0.121	**0.007**

**AFP (ng/mL)**	5.9	5.5	5.8	5.4
	(0.8 - 71221.0)	(0.9 - 73955.0)	(0.9 - 82861.0)	(0.9 - 70228.0)
		0.032	0.086	0.553

**CYFRA 21-1 (ng/mL)**	1.8	1.7	1.6	2.0
	(0.5 - 20.9)	(0.5 - 21.6)	(0.5 - 22.1)	(0.6 - 31.0)
		0.702	**0.017**	**0.003**

**LDH (U/L)**	200.0	199.0	204.5	240.0
	(139.0 - 403.0)	(137.0 - 412.0)	(136.0 - 391.0)	(155.0 - 1062.0)
		0.958	0.351	**< 0.001**

**CRP (mg/dL)**	0.5	0.5	0.6	1.4
	(0.1 - 10.5)	(0.1 - 11.7)	(0.1 - 12.4)	(0.1 - 13.3)
		0.802	0.262	**< 0.001**

**AST (U/L)**	49.0	51.0	53.5	90.0
	(18.0 - 238.0)	(20.0 - 263.0)	(18.0 - 248.0)	(30.0 - 710.0)
		0.063	**< 0.001**	**< 0.001**

**ALT (U/L)**	38.0	38.0	40.0	60.0
	(12.0 - 179.0)	(15.0 - 190.0)	(16.0 - 198.0)	(21.0 - 740.0)
		0.083	0.313	**< 0.001**

**GLDH (U/L)**	6.6	5.9	6.5	16.8
	(1.0 - 42.3)	(1.0 - 36.2)	(1.0 - 33.9)	(1.5 - 206.0)
		0.065	0.574	**< 0.001**

**Bilirubin (mg/dL)**	0.9	1.2	1.2	1.5
	(0.3 - 6.1)	(0.3 - 7.3)	(0.3 - 7.2)	(0.5 - 9.5)
		**< 0.001**	**< 0.001**	**< 0.001**

**GGT (U/L)**	156.0	153.0	161.0	168.0
	(25.0 - 1007.0)	(22.0 - 888.0)	(23.0 - 911.0)	(25.0 - 930.0)
		**< 0.001**	0.092	**0.001**

**AP (U/L)**	128.0	113.0	124.0	124.0
	(58.0 - 421.0)	(57.0 - 408.0)	(54.0 - 416.0)	(62.0 - 468.0)
		**< 0.001**	**< 0.001**	0.221

**Amylase (U/L)**	82.0	80.0	81.5	77.0
	(24.0 - 839.0)	(19.0 - 773.0)	(20.0 - 821.0)	(24.0 - 829.0)
		**< 0.001**	0.322	0.131

**Lipase (U/L)**	42.0	36.0	36.5	34.0
	(9.0 - 342.0)	(8.0 - 149.0)	(6.0 - 127.0)	(8.0 - 188.0)
		**< 0.001**	0.061	**< 0.001**

**CHE (kU/L)**	4.4	4.4	4.5	4.4
	(0.9 - 8.8)	(0.8 - 9.4)	(0.9 - 8.8)	(0.9 - 9.7)
		0.563	0.840	**0.002**

**Urea (mg/dL)**	33.0	33.0	35.5	35.0
	(16.0 - 87.0)	(17.0 - 79.0)	(16.0 - 81.0)	(17.0 - 89.0)
		0.079	0.316	**0.014**

**Uric acid (mg/dL)**	5.9	6.2	6.1	6.0
	(3.2 - 10.7)	(3.2 - 10.9)	(3.4 - 10.9)	(3.5 - 10.9)
		**0.011**	**0.018**	0.438

**Creatinine (mg/dL)**	0.9	0.9	1.0	1.0
	(0.6 - 1.6)	(0.5 - 1.7)	(0.5 - 1.7)	(0.6 - 1.8)
		**0.001**	0.393	0.201

### Correlation with therapy response

Although pretherapeutic values of circulating nucleosomes were not different in both responder groups, higher values of circulating nucleosomes were measured 24 h after treatment in patients with progressive disease compared to non progressive patients (p = 0.002). In consequence, the percental changes of circulating nucleosomes between pretherapeutic values and 24 h values were significantly greater in the progressive group (p < 0.001). Interestingly, 3 h and 6 h values were temporarily decreased in many patients.

**Figure 2:**
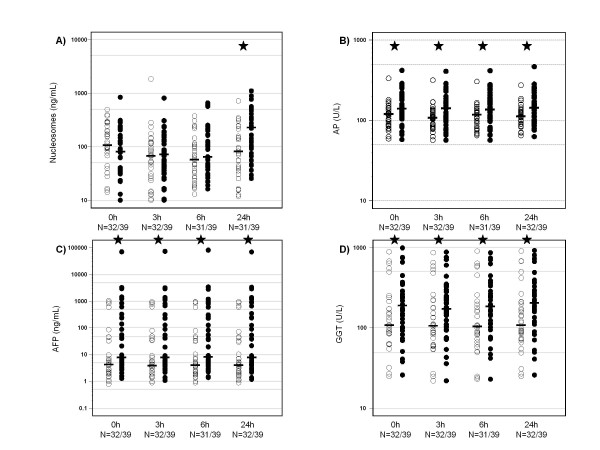
**Dot-plots of serum levels of nucleosomes, alkaline phosphatase (AP), alpha fetoprotein (AFP) and gamma-glutamyl-transferase (GGT) with respective medians of patients with response to therapy (○) and those with no response to therapy (●) for all time points during the first day after TACE (★ indicate significant differences)**.

The other two cell death parameters LDH and CYFRA 21-1 were increased 24 h after treatment with no significant differences between both groups. However, 3 h and 6 h after TACE, CYFRA 21-1 values showed slightly significant differences (p = 0.041). Just as nucleosomes, CYFRA 21-1 showed decreasing values in both groups after 3 h and 6 h, whereby responders had decreasing levels more often than non-responders. Further markers which differed in the two response groups before and 24 h after TACE were AFP, GGT, AP as well as ALT 3 h and 6 h after TACE (Table 3 and Figure 2).

**Table 3:** Correlation of biomarkers with therapy response

Marker	Time	Response N = 32	No-Response N = 39	P-Value
**Nucleosomes (ng/mL)**	0 h	107.5	80.7	0.142
	24 h	81.8	231.1	**0.002**
	pc.	-26.2	114.3	**< 0.001**

**AFP (ng/mL)**	0 h	4.4	7.9	**0.011**
	24 h	4.2	7.9	**0.010**
	pc.	0	0	0.822

**CYFRA 21-1 (ng/mL)**	0 h	1.8	2.0	0.349
	24 h	2.0	2.2	0.101
	pc.	0	14.3	0.229

**LDH (U/L)**	0 h	198.5	200.0	0.288
	24 h	240.0	250.0	0.746
	pc.	21.6	21.0	0.712

**CRP (mg/dL)**	0 h	0.4	0.8	0.424
	24 h	1.2	1.5	0.361
	pc.	100.0	133.3	0.644

**CHE (kU/L)**	0 h	4.4	4.4	0.636
	24 h	4.6	4.4	0.652
	pc.	3.0	4.6	0.514

**AST (U/L)**	0 h	41.5	56.0	0.084
	24 h	101.0	89.0	0.556
	pc.	102.6	50.0	0.241

**ALT (U/L)**	0 h	37.5	38.0	0.652
	24 h	63.5	57.0	0.138
	pc.	72.6	19.8	0.051

**GLDH (U/L)**	0 h	5.0	7.7	0.150
	24 h	12.7	18.4	0.922
	pc.	151.7	90.0	0.280

**Bilirubin (mg/dL)**	0 h	0.9	1.0	0.276
	24 h	1.5	1.6	0.231
	pc.	47.7	60.0	0.995

**GGT (U/L)**	0 h	109.0	192.0	**0.034**
	24 h	108.5	206.0	**0.027**
	pc.	2.8	5.7	0.234

**AP (U/L)**	0 h	119.5	140.0	**0.023**
	24 h	112.5	143.0	**0.006**
	pc.	-4.1	1.9	0.317

Median values and p-values (Wilcoxon-test) are shown for the two response groups (pc. = percental changes)

Concerning clinical factors, T1 and T2 tumors were associated with better response to therapy when compared to T3 stage disease (p = 0.191). Also, the number of TACE cycles correlated with poor response (p = 0.0266). Importantly, the number of TACE cycles did not correlate with levels of markers associated with treatment response, particularly nucleosomes, AFP, GGT, AP and ALT. Notably, therapy response correlated with overall survival of the patients as well underlining its relevance as a meaningful endpoint (p = 0.019).

**Figure 3:**
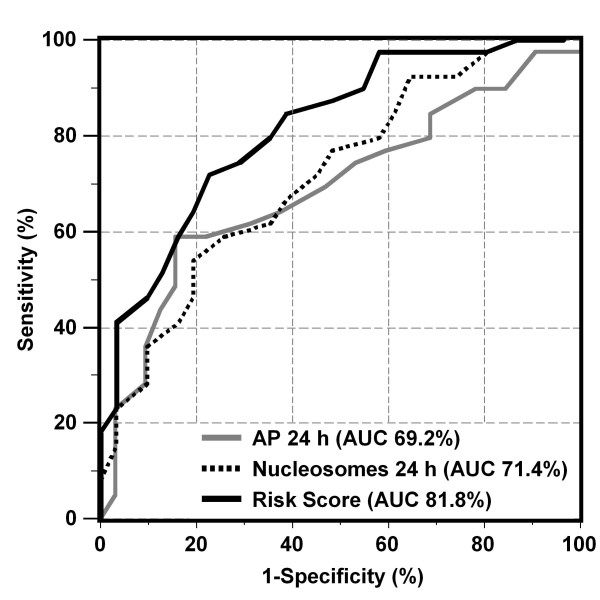
**Receiver operating characteristic (ROC) curves indicating the profile of sensitivity and specificity for the estimation of non-response to therapy over the whole range of possible cutoffs for nucleosomes (24 h), alkaline phosphatase (AP 24 h) and the risk score of the combination of both markers and the number of TACE cycle that was identified as best predictive model in multivariate analysis**.

All univariately relevant biochemical markers measured before (0 h) and 24 h after TACE indicating later therapy response were logarithmized and included, together with clinical factors, into a multivariate logistic regression analysis. Next, the best model of independent predictive markers was identified as combination of nucleosomes (24 h), AP (24 h) and TACE cycle number (Table 4). A similar predictive strength, objectified by Akaike information criterion, was achieved when AP (24 h) was replaced by AFP (24 h). The area under the curve (AUC) of the receiver operating characteristic (ROC) curves for estimation of non-response to therapy was considerably improved when using the risk score based on the combination model (AUC 81.8%) as compared to the single marker nucleosomes (24 h; 71.4%) and AP (24 h; 69.2%; Figure 3). The resulting sensitivities for prediction of non-response to therapy were 41% at 97% specificity, and 72% at 78% specificity using the risk score.

**Table 4:** Best multivariate model of independent predictive biochemical and clinical markers for poor efficacy of TACE therapy

Parameter	Coefficient	Odds-Ratio	95%-Conf. Interval	Chi-Square	P-value
**Nucleosomes** (24 h, log2)	**0.581**	**1.79**	1.20 - 2.66	**8.25**	**0.0041**

**Alkaline** **Phosphatase** (24 h, log2)	**1.561**	**4.76**	1.45 - 15.64	**6.62**	**0.0101**

**Number of** **TACE cycle**	**0.636**	**1.89**	1.18 - 3.02	**7.08**	**0.0078**

## Discussion

Diagnosis of hepatocellular carcinoma is often only performed in advanced stages of the disease due to the late appearance of symptoms. In recent years, the introduction of screening programs in patients with major risk factors has resulted in earlier diagnosis of HCC. However, many patients are still diagnosed at a late stage when treatment in curable intention is no longer an option [6,25]. In HCC patients considered unsuitable candidates for surgery or ablative therapies, TACE is the most widely used treatment option and it has recently been shown to improve survival in comparison to best supportive care [8-13].

It is known that in tumor tissue, massive cell proliferation and cell death often coexist at the same time. This turnover of cells, including the release of apoptotic products like circulating nucleosomes, varies between different kinds of cancers and also between individuals [26]. Also in hepatocellular carcinoma, dysregulation of the balance between proliferation and cell death has been found. Downregulation of proapoptotic molecules, such as p53, and upregulation of antiapoptotic markers, such as NF-κB, may - inter alia - be responsible for this imbalance [27]. Nevertheless, the apoptosis index was found to be higher in advanced tumor stages and in patients with metastasis. This is possibly due to the increasing amount of dysfunctional cells [28]. As HCC is often diagnosed in advanced stages and circulating nucleosomes are markers for cell death, we expected to find high rates of nucleosomes pretherapeutically as well as during therapy. The pretherapeutic median value of nucleosomes was higher than in healthy individuals (36 ng/mL) [29]. Subsequently, values temporarily declined in most patients 3 h and 6 h after therapy, followed by a rapid increase after 24 hours. However, comparison of patients with T-stages 1 and 2 disease versus T3-stage disease revealed no significant difference between both groups. This is in line with earlier findings in pancreatic and colorectal cancer during systemic radiochemotherapy and may be explained by cell cycle arrest and repair processes which occur during the early phase after cellular and chromatin damages [30,31]. Furthermore, the cell death biomarkers CYFRA 21-1 and LDH, the liver enzymes ALT, AST, and GLDH as well as cholestasis parameters such as bilirubin and GGT also increased 24 hours after therapy.

As macroscopic changes in tumor volume often occur only some time after the application of cytotoxic therapies and as their measurement is cost-intensive, biochemical diagnostic tools are required to provide an earlier and less expensive estimation of therapy efficacy and to enable, if possible, effective modification or intensification of therapy, e.g. with sorafenib or other targeted agents [32]. In our setting, indication of therapy response by circulating nucleosomes measured after 24 h was highly significant. Responders to therapy had decreasing levels of circulating nucleosomes 24 h after chemoembolization, while non-responders showed increasing levels. In consequence, percental changes of circulating nucleosomes pretherapeutically and 24 h after TACE were significantly different. It can be speculated that the observed increases of circulating nucleosome levels in patients with no response to chemotherapy is caused by the presence of more aggressive and larger tumors. Furthermore, the efficiency of the local immune system may be limited in patients with advanced tumors leading to a delayed local and plasmatic elimination of liberated nucleosomes [29].

Interestingly, several biochemical liver parameters showed significant differences in the response groups: AFP, AP and GGT were elevated before and 24 h after TACE in patients who suffered from less efficient therapies.

Some patients were enrolled not at their first but at a later TACE treatment. As it was observed that first or second applications of TACE were associated with better outcome when compared with later therapies, influence of cycle number on biomarker values was assessed. However, at least for the meaningful predictive markers, no association with treatment cycle number was found.

As several biomarkers measured before (0 h) and 24 h after TACE have been identified to indicate later therapy response in univariate analyses, they were included with clinical factors in a multivariate logistic regression analysis. Thus, the combination of nucleosomes (24 h), AP (24 h) and TACE cycle number was found to be the best model of independently predictive markers, while replacement of AP with AFP led to quite similar results. It has to be pointed out that markers were included into the model on a logarithmic scale to the basis 2 in order to avoid too optimistic cutoff definitions and overfitting to the sample. In consequence, any doubling of the marker values was associated with a relative risk of 1.79 for nucleosomes and 4.76 for AP for non-response to therapy.

Using the risk score based on the proposed combination model, the AUC in ROC curves and the respective sensitivities at defined specificities were considerably improved in comparison with the use of only single markers. Notably, a sensitivity of 41% for prediction of non-response to therapy was obtained at 97% specificity, and a 72% sensitivity at 78% specificity. As TACE cycle numbers were included into the multivariate model this would be applicable independently regardless whether the first or a later TACE treatment is applied.

Obviously, the present study has some drawbacks, such as the limited number of patients and the scatter in the intervals of determining therapy response. Although most patients had been screened prior to their next TACE treatment or at radiological control exams 60 to 90 days after TACE treatment, some exceptions ocurred, such as the case of one patient who died 22 days after TACE due to the cancer disease. This patient was therefore classified as non-responder. Another patient did not return to hospital due to his good physical condition and was only staged after 216 days. In total, there were four patients with staging intervals of more than four months. Two of these patients responded well to therapy.

It has to be emphasized that all patients were included in the study prospectively and with no other than the indicated selection criteria in order to enable narrow-meshed blood sampling, control of the preanalytics and to guarantee completeness of data.

## Conclusions

Overall, our results demonstrate the high relevance of circulating nucleosomes and other liver markers in addition to clinical parameters for early estimation of the later response to TACE therapy in HCC patients. The model of nucleosomes (24 h), AP (24 h) and TACE number is recommended for further validation in clinical trials.

## Authors' contributions

NK, DJ, PS, DN and SH designed the present study and coordinated the logistic process. NK, DJ and JD were responsible for recruitment of patients, defined blood sampling and clinical data collection. NK, PS and SH were responsible for immunoassay measurements. Statistical analysis was performed by DN. NK, PS, DN and SH were involved in the interpretation of the data and the conception of the manuscript. All authors (except DJ who died during the study) read and approved the final manuscript.

## Pre-publication history

The pre-publication history for this paper can be accessed here:

http://www.biomedcentral.com/1471-2407/11/202/prepub

## References

[B1] Parkin DM Bray F Ferlay J Pisani P Estimating the world cancer burden: GLOBOCAN 2000 Int J Cancer 2001 94 153 156 10.1002/ijc.1440 11668491

[B2] El-Serag HB Mason AC Hepatocellular carcinoma is rising in the United States Gastroenterol 1999 116 G0235 10.1016/S0016-5085(99)70115-0 10072408

[B3] Hellerbrand C Hartmann A Richter G Knoll A Wiest R Scholmerich J Lock G Hepatocellular carcinoma in southern Germany: Epidemiological and clinicopathological characteristics and risk factors Digest Dis 2001 19 345 351 10.1159/000050702 11935095

[B4] Tsukuma H Hiyama T Tanaka S Nakao M Yabuuchi T Kitamura T Nakanishi K Fujimoto I Inoue A Yamazaki H Kawashima T Risk factors for hepatocellular carcinoma among patients with chronic liver disease N Engl J Med 1993 328 1797 1801 10.1056/NEJM199306243282501 7684822

[B5] Velazquez RF Rodriguez M Navascues CA Linares A Perez R Sotorrios NG Martinez I Rodrigo L Prospective analysis of risk factors for hepatocellular carcinoma in patients with liver cirrhosis Hepatol 2003 37 520 527 10.1053/jhep.2003.50093 12601348

[B6] El-Serag HB Marrero JA Rudolph L Reddy KR Diagnosis and treatment of hepatocellular carcinoma Gastroenterol 2008 134 1752 1763 10.1053/j.gastro.2008.02.090 18471552

[B7] Forner A Hessheimer AJ Real MI Bruix J Treatment of hepatocellular carcinoma Crit Rev Oncol Hematol 2006 60 89 98 10.1016/j.critrevonc.2006.06.001 16860993

[B8] Bruix J Llovet JM Castells A Montana X Bru C Ayuso MD Vilana R Rodes J Transarterial embolization versus symptomatic treatment in patients with advanced hepatocellular carcinoma: Results of a randomized, controlled trial in a single institution Hepatol 1998 27 1578 1583 10.1002/hep.510270617 9620330

[B9] Llovet JM Bruix J Systematic review of randomized trials for unresectable hepatocellular carcinoma: Chemoembolization improves survival Hepatol 2003 37 429 442 10.1053/jhep.2003.50047 12540794

[B10] Llovet JM Real MI Montana X Planas R Coll S Aponte J Ayuso C Sala M Muchart J Sola R Rodes J Bruix J Arterial embolisation or chemoembolisation versus symptomatic treatment in patients with unresectable hepatocellular carcinoma: a randomised controlled trial Lancet 2002 359 1734 1739 10.1016/S0140-6736(02)08649-X 12049862

[B11] Anonymous A comparison of lipiodol chemoembolization and conservative treatment for unresectable hepatocellular carcinoma. Groupe d'Etude et de Traitement du Carcinome Hépatocellulaire N Engl J Med 1995 332 1256 1261 7708069 10.1056/NEJM199505113321903

[B12] Pelletier G Ducreux M Gay F Luboinski M Hagege H Dao T Van Steenbergen W Buffet C Rougier P Adler M Pignon JP Roche A Treatment of unresectable hepatocellular carcinoma with lipiodol chemoembolization: a multicenter randomized trial J Hepatol 1998 29 129 134 10.1016/S0168-8278(98)80187-6 9696501

[B13] Takayasu K Arii S Ikao I Omata M Okita K Ichida T Matsuyama Y Nakanuma Y Kojiro M Makuuchi M Yamaoka Y Prospective cohort study of transarterial chemoembolization for unresectable hepatocellular carcinoma in 8510 patients Gastroenterol 2006 131 461 469 10.1053/j.gastro.2006.05.021 16890600

[B14] Bruix JSM Llovet JM Chemoembolization for hepatocellular carcinoma Gastroenterol 2004 127 179 188 10.1053/j.gastro.2004.04.006 15508083

[B15] Lim HS Jeong YY Kang HK Kim JK Park JG Imaging features of hepatocellular carcinoma after transcatheter arterial chemoembolization and radiofrequency ablation Am J Roentgenol 2006 187 W341 349 10.2214/AJR.04.1932 16985104

[B16] Takayasu K Arii S Matsuo N Yoshikawa M Ryu M Takasaki K Sato M Yamanaka N Shimamura Y Ohto M Comparison of CT findings with resected specimens after chemoembolization with iodized oil for hepatocellular carcinoma Am J Roentgenol 2000 175 699 704 10.2214/ajr.175.3.1750699 10954453

[B17] Thabet A Kalva S Gervais DA Percutaneous image-guided therapy of intra-abdominal malignancy: imaging evaluation of treatment response Abdom Imaging 2009 34 593 609 10.1007/s00261-008-9448-9 19015913

[B18] Chen CY Li CW Kuo YT Jaw TS Wu DK Jao JC Hsu JS Liu GC Early response of hepatocellular carcinoma to transcatheter arterial chemoembolization: Choline levels and MR diffusion constants initial experience Radiol 2006 239 448 456 10.1148/radiol.2392042202 16569781

[B19] Kamel IR Bluemke DA Eng J Liapi E Messersmith W Reyes DK Geschwind JFH The role of functional MR imaging in the assessment of tumor response after chemoembolization in patients with hepatocellular carcinoma J Vasc Intervent Radiol 2006 17 505 512 10.1097/01.RVI.0000200052.02183.92 16567675

[B20] Holdenrieder S von Pawel J Dankelmann E Duell T Faderl B Markus A Siakavara M Wagner H Feldmann K Hoffmann H Raith H Nagel D Stieber P Nucleosomes and CYFRA 21-1 indicate tumor response after one cycle of chemotherapy in recurrent non-small cell lung cancer Lung Cancer 2009 63 128 135 10.1016/j.lungcan.2008.05.001 18571761

[B21] Holdenrieder S v Pawel J Dankelmann E Duell T Faderl B Markus A Siakavara M Wagner H Feldmann K Hoffmann H Raith H Nagel D Stieber P Nucleosomes, ProGRP, NSE, CYFRA 21-1 and CEA in the therapy monitoring of small-cell lung cancer during first-line chemotherapy Clin Cancer Res 2008 14 7813 7821 10.1158/1078-0432.CCR-08-0678 19047109

[B22] Bruix J Sherman M Llovet JM Beaugrand M Lencioni R Burroughs AK Christensen E Pagliaro L Colombo M Rodes J Clinical management of hepatocellular carcinoma. Conclusions of the Barcelona-2000 EASL Conference J Hepatol 2001 35 421 430 10.1016/S0168-8278(01)00130-1 11592607

[B23] Therasse P Arbuck SG Eisenhauer EA Wanders J Kaplan RS Rubinstein L Verweij J Van Glabbeke M van Oosterom AT Christian MC Gwyther SG New guidelines to evaluate the response to treatment in solid tumors J Natl Cancer Inst 2000 92 205 216 10.1093/jnci/92.3.205 10655437

[B24] Holdenrieder S Stieber P Bodenmuller H Fertig G Furst H Schmeller N Untch M Seidel D Nucleosomes in serum as a marker for cell death Clin Chem Lab Med 2001 39 596 605 10.1515/CCLM.2001.095 11522104

[B25] Ikai I Arii S Kojiro M Ichida T Makuuchi M Matsuyama Y Nakanuma Y Okita K Omata M Takayasu K Yamaoka Y Reevaluation of prognostic factors for survival after liver resection in patients with hepatocellular carcinoma in a Japanese nationwide survey Cancer 2004 101 796 802 10.1002/cncr.20426 15305412

[B26] Meyn RE Stephens LC Ang KK Hunter NR Brock WA Milas L Peters LJ Heterogeneity in the development of apoptosis in irradiated murine tumors of different histologies Int J Radiat Biol 1993 64 583 591 10.1080/09553009314551801 7902398

[B27] Fabregat I Dysregulation of apoptosis in hepatocellular carcinoma cells World J Gastroenterol 2009 15 513 520 10.3748/wjg.15.513 19195051 PMC2653340

[B28] Jing Z Nan KJ Hu ML Cell proliferation, apoptosis and the related regulators p27, p53 expression in hepatocellular carcinoma World J Gastroenterol 2005 11 1910 1916 15800979 10.3748/wjg.v11.i13.1910 PMC4305710

[B29] Holdenrieder S Stieber P Clinical use of circulating nucleosomes Crit Rev Clin Lab Sci 2009 46 1 24 10.1080/10408360802485875 19107649

[B30] Kremer A Wilkowski R Holdenrieder S Nagel D Stieber P Seidel D Nucleosomes in pancreatic cancer patients during radiochemotherapy Tumor Biol 2005 26 44 49 10.1159/000084339 15756056

[B31] Kremer A Holdenrieder S Stieber P Wilkowski R Nagel D Seidel D Nucleosomes in colorectal cancer patients during radiochemotherapy Tumor Biol 2006 27 235 242 10.1159/000094694 16864976

[B32] Beachy SH Repasky EA Using extracellular biomarkers for monitoring efficacy of therapeutics in cancer patients: an update Cancer Immunol Immunother 2008 57 759 775 10.1007/s00262-007-0445-6 18188561 PMC11029872

